# From Brownian motion to virtual biopsy: a historical perspective from 40 years of diffusion MRI

**DOI:** 10.1007/s11604-024-01642-z

**Published:** 2024-09-18

**Authors:** Denis Le Bihan

**Affiliations:** 1 NeuroSpin, CEA, Paris-Saclay University, Bât 145, CEA-Saclay Center, 91191 Gif-sur-Yvette, France; 2 Human Brain Research Center, Kyoto University, Kyoto, Japan; 3 Department of System Neuroscience, National Institutes for Physiological Sciences, Okazaki, Japan

**Keywords:** MRI, Diffusion MRI, DTI, Tractography, Oncology, Neurology, Psychiatry

## Abstract

Diffusion MRI was introduced in 1985, showing how the diffusive motion of molecules, especially water, could be spatially encoded with MRI to produce images revealing the underlying structure of biologic tissues at a microscopic scale. Diffusion is one of several Intravoxel Incoherent Motions (IVIM) accessible to MRI together with blood microcirculation. Diffusion imaging first revolutionized the management of acute cerebral ischemia by allowing diagnosis at an acute stage when therapies can still work, saving the outcomes of many patients. Since then, the field of diffusion imaging has expanded to the whole body, with broad applications in both clinical and research settings, providing insights into tissue integrity, structural and functional abnormalities from the hindered diffusive movement of water molecules in tissues. Diffusion imaging is particularly used to manage many neurologic disorders and in oncology for detecting and classifying cancer lesions, as well as monitoring treatment response at an early stage. The second major impact of diffusion imaging concerns the wiring of the brain (Diffusion Tensor Imaging, DTI), allowing to obtain from the anisotropic movement of water molecules in the brain white-matter images in 3 dimensions of the brain connections making up the Connectome. DTI has opened up new avenues of clinical diagnosis and research to investigate brain diseases, neurogenesis and aging, with a rapidly extending field of application in psychiatry, revealing how mental illnesses could be seen as Connectome spacetime disorders. Adding that water diffusion is closely associated to neuronal activity, as shown from diffusion fMRI, one may consider that diffusion MRI is ideally suited to investigate both brain structure and function. This article retraces the early days and milestones of diffusion MRI which spawned over 40 years, showing how diffusion MRI emerged and expanded in the research and clinical fields, up to become a pillar of modern clinical imaging.

## Introduction

Diffusion MRI was born in the mid-1980s. Since then, it has enjoyed incredible success over the past 40 years, both for research and in the clinical field. Clinical applications began in the brain, notably in the management of acute stroke patients. Diffusion MRI then became the standard for the study of cerebral white-matter diseases, through the diffusion tensor imaging (DTI) framework, revealing abnormalities in the integrity of white-matter fibers in neurologic disorders and, more recently, mental disorders. Over time, clinical applications of diffusion MRI have been extended, notably in oncology, to diagnose and monitor cancerous lesions in almost all organs of the body [1]. Diffusion MRI has become a reference-imaging modality for prostate and breast cancer [2, 3]. Diffusion MRI began in my hands in 1984 (I was then a radiology resident and a PhD student in nuclear and particle physics) with my intuition that measuring the molecular diffusion of water would perhaps allow to characterize solid tumors due to the restriction of molecular motion and vascular lesions where in circulating blood “diffusion” would be somewhat enhanced. This idea was to become the cornerstone of diffusion MRI. This article retraces the early days and milestones of diffusion MRI which spawned over 40 years.

## The scaling concept beyond diffusion MRI

In his 1905 doctoral thesis (dated April 30th, 1905, and a companion article which is his most cited, more than his article on the relativity theory published one month later), Einstein established a link between diffusion (a macroscopically visible phenomenon, for instance when one pours ink in a glass of water) and Brownian motion (a microscopic phenomenon visible only under a microscope) in the context of the theory of heat and energy, to demonstrate the existence of atoms and molecules [4]. Brownian motion was discovered in 1827 by the botanist Robert Brown who, using a microscope, observed the spontaneous, random movement of pollen grains suspended in water, but was unable to identify the mechanism behind this movement (he even saw in it the secret of life!). On the other hand, the macroscopic phenomenon of “diffusion”—the net movement of solutes from a region of high concentration to a region of low concentration—was mathematically described by Adolf Fick in a series of equations in 1855. Einstein intuitively showed that, if molecules exist, particles in suspension who receive the pressure of underlying individual molecules, would exhibit random displacements quantitatively related to the diffusion coefficient of Fick’s law, thus somehow predicting (or rediscovering) Brownian motion from a theoretical standpoint, without doing a single experiment. This clever thought was for the first time bridging the gap between the concepts of diffusion and Brownian motion, and thus between the macroscopic and the microscopic scales. Amazingly, decades later, MRI allowed to obtain images of the diffusion process of water molecules in the human body completely non-invasively. The guiding concept of diffusion MRI, which explains its great success, is to reveal at a *macroscopic* scale (the usual millimeter resolution of MRI) the underlying phenomena occurring at a *microscopic* scale: diffusing water molecules encounter tissue structures and probe them at micrometer level. In some way, diffusion MRI is a kind of virtual biopsy.

More precisely, the Brownian motion distance of freely and randomly diffusing molecules is statistically well described by a random walk model and linked to the “diffusion coefficient”, *D,* of Fick. The statistical, mean-squared distance traveled by diffusing molecules *along one spatial direction* (diffusion is, in fact, a tridimensional process) in a given interval of time, *T*_d_, (diffusion time) is:

1 $$< X^{2} > \, = \,2DT_{d}$$

where < *X*^*2*^ > is the average mean-squared diffusion distance along this direction (in 3D one has to replace 2 by 6). Given that the (free) diffusion coefficient of water at the body temperature is around 3.10^–9^ m^2^ s^−1^ Eq. (1) means that about 32% of the molecules have reached at least 17 μm during 50 ms (typical diffusion time for diffusion MRI). This distance depends only on D which, in turn, depends on the size (mass) of the molecules (this allowed Perrin to estimate the size of the water molecule from Brownian motion in 1908 and to receive the Nobel Prize in 1926), the temperature and the nature (viscosity) of the medium. However, if viscosity was the only mechanism for water diffusion, diffusion MRI would not have gone very far, resulting in poor contrast between tissues (but diffusion MRI can be used to estimate tissue temperature, for instance during hyperthermia session [5, 6]).

Indeed, in tissues, the actual diffusion distance is much shorter, as water molecules must bounce, cross, contour or interact with many tissue components, such as cell membranes, fibres or macromolecules. It is this non-invasive observation with diffusion MRI of such water hindered diffusion-driven displacement distributions in vivo at a microscopic scale which provides us with a unique contrast, giving us clues to the fine structural features and geometric organization of tissues, and to the variations in those features according to changes in physiologic or pathologic states.

Another important parameter in Eq. (1) is the diffusion time, *T*_d_. At very short times diffusion molecules do not have enough time to interact with tissues components, their displacements being mainly driven by the local intrinsic viscosity. When diffusion times become longer the effects of obstacles become predominant, impacting molecular diffusion displacements. In the early days of diffusion MRI the gradient hardware used to encode diffusion displacements was rather weak, which imposed long diffusion times (> 70 ms) to produce sufficient diffusion encoding. Luckily, this limitation was a blessing, allowing, surprisingly, great contrast to be seen between tissues, which contributed to the success of diffusion MRI. With shorter diffusion times, contrast would have been dulled and diffusion MRI might have been still born. Nowadays, however, with strong gradient hardware, short diffusion times are reachable, showing that, nonetheless, useful information can be retrieved by observing tissue-dependant changes in water diffusion behavior over diffusion times (see below).

## From NMR artifact to MRI stardom

Diffusion was first identified as a nuisance soon after the introduction of NMR (Nuclear Magnetic Resonance) by Bloch and Purcell (Nobel Prize 1942). Because of the poor field homogeneity of magnets in the 1950s, diffusing molecules encountered variations in field strength which broadened and weakened NMR signals. Pioneers such as Hahn, Carr, Torrey and others worked out theories to explain how diffusion in magnetic field gradients would impact NMR signals and how diffusion effects could be mitigated by using ad-hoc NMR sequences. Stejskal and Tanner changed the paradigm in the 1960s by suggesting (and showing) that NMR could be used instead to investigate diffusion, notably in (dead) biologic tissues. To do so, they devised a specific gradient pulse scheme, the famous Stejskal-Tanner sequence, which had the merit to well define the diffusion time [7]. However, an obvious, but important limitation was that NMR did not allow their measurements to be localized: only diffusion coefficients from the whole investigated samples could be obtained. Localization of diffusion within samples, furthermore in vivo, became possible in the 1980s, after the advent of MRI.

The story is that I started thinking about making images of diffusion in 1984 to address a challenge that Professor Grenier (one member of the teams of radiologists with whom we were evaluating the clinical potential of MRI using the “Magniscan’ 0.35 T MRI scanner from the then “Compagnie Générale de Radiologie” (CGR, a French company located in Buc near Versailles in France, acquired by General Electric Medical Systems (GEMS) in 1987) had given to me: Would it be possible with MRI to differentiate liver tumors from angiomas? There were no MRI contrast media clinically available at that time. As I mentioned above I thought that molecular diffusion measurements of water could be the solution. Wesbey et al. [8] had just shown in a phantom made of water and acetone that diffusion had a detectable effect in MRI scans and that such effect could be modulated by modifying some of the acquisition parameters (notably the amplitude of the slice selection gradient which, obviously, also changed in slice thickness, making it unpractical for in vivo applications). Hence, it was clear to me that localized and accurate measurements of diffusion in tissues would require specific treatment. The Stejskal-Tanner pulse scheme was appealing, but the problem was to mix such pulses with those used in the MRI sequence for spatial encoding and to quantity the overall resulting effect. Clearly the equation given by Stejskal and Tanner in their seminal article [7] could no longer work to get correct measurements of diffusion. The novelty was to *localize* the diffusion measurements on a voxel basis, that is to obtain *accurate quantitative maps* of the diffusion coefficients in tissues, which had never been done before, especially in vivo, with any technique. I was very excited and, in a matter of weeks “diffusion MRI”, as we know it and still use it today, was born, implemented and patented. It was not trivial, several technical issues had to be worked out, to the extent that many considered at the time that diffusion MRI was not clinically possible.

**Fig. 1:**
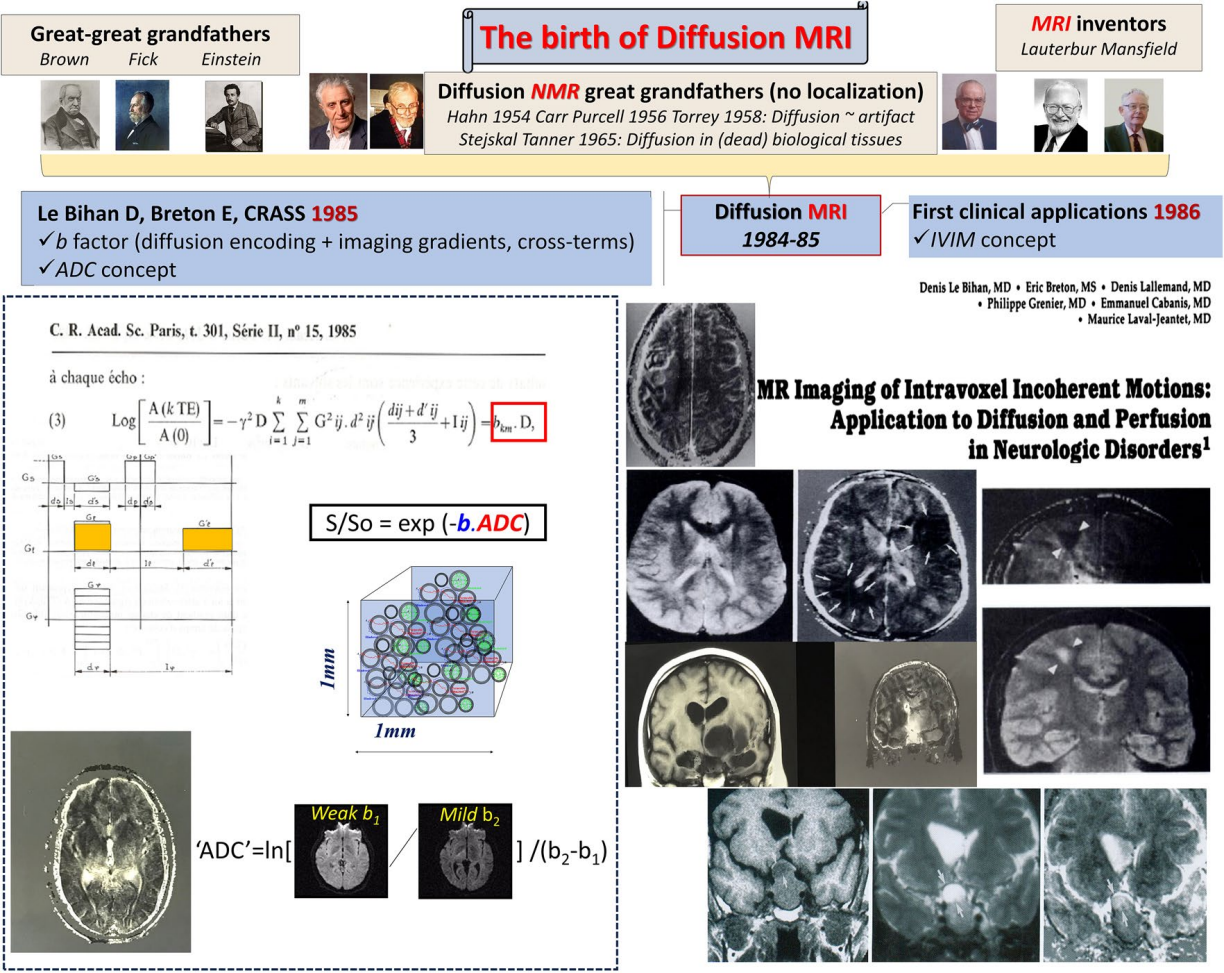
The birth of Diffusion MRI. Left: The ingredients of diffusion MRI, as we know it today, were introduced in an article published in French in the Compte Rendus de l’Académie des Sciences (1985) [9]. The article introduces the diffusion MRI sequence, based on the Stejskal-Tanner sequence, but shows how diffusion effects must include combined effects of diffusion-encoding and imaging gradient pulses (so-called b factor). The resulting images calculated from the acquisition of 2 sets of diffusion-weighted images are called “ADC maps” (Apparent Diffusion Coefficient) underlying that the diffusion mechanisms at play at voxel levels are complex and do not reflect free diffusion. Right: First clinical diffusion images shown in a scientific exhibit presented at the RSNA meeting in 1985 and published in Radiology in 1986 [13]. Examples taken from a small cohort of pediatric neurology patients (astrocytoma, tumor with edema, chronic stroke, cystic tumor) showed a new type of contrast, not seen with T1/T2 relaxation, including CSF flow effects

The first article on diffusion MRI, as we know it today, was published in 1985 in French in the journal of the Academy of Sciences [9], contributed by the legendary Anatole Abragam, a source of great pride for me. In this article I introduced all the ingredients which are used today for diffusion MRI, especially the basic diffusion MRI equation (Fig. 1):

2 $$S/So = {\text{exp }}\left( { - bD} \right)$$

S/So is the diffusion-driven signal attenuation, *D* the Diffusion Coefficient and *b* is the famous “*b*” factor (which I took from my name “B”ihan) to avoid startling radiologists with a scary double integral equation. Indeed, the Stejskal-Tanner equation had to be replaced by a much more complex one, considering the contribution of both the gradient pulses used for sensitizing the MRI sequence to diffusion and the imaging gradient pulses, but also and notably the cross-terms between all those gradients which are far from negligible (to my dismay some groups are still citing the Stejskal-Tanner article paper in the Method section of their articles in the context of quantitative diffusion MRI, although it is not appropriate and not used by vendors to calculate the b values of their sequences).

As it was my challenge, my first attempts were made in the liver but the results were disappointing, because of huge motion artifacts from respiration (respiratory gating was not available and motion artifacts were atrocious in the body). Interestingly, Yamada et al. [10] demonstrated many years later that my idea to separate angiomas from liver tumors with diffusion MRI was correct. Also, the signal-to-noise ratio was very low at 0.35 T and the gradient hardware barely allowed strengths beyond 8 or 10 mT/m (corresponding to b values no larger than 100 or 200 mm/s^2^, a tenth of the values available today on any standard MRI scanner). Gradient coils were generating strong eddy-currents, leading to severe artifacts. Luckily, I quickly switched to the brain (I started my residency as a neurosurgeon before converting to neuroradiology), a much less mobile organ, and the results were very encouraging. After scanning my own brain and those of some of my colleagues (a common practice at the time) the first results obtained in a cohort of pediatric neurosurgery patients were stunning. Clearly, a new imaging modality was born: Diffusion MRI (at least in my mind, it would take many more years for diffusion MRI to enter clinical practice, see below). I presented the world’s first diffusion images of the brain in an oral presentation at the 1985 meeting of the (International) Society of Magnetic Resonance in Medicine, (I)SMRM, in London. Those images were quantitative, which was not the case for the images of the hand shown at the same meeting by Dieter Merboldt using a diffusion-sensitized stimulated-echo sequence [11]. The only other presentation (poster) regarding diffusion was made by Taylor and Bushel who showed diffusion measurements in a chicken egg using a steady gradient pulse without considering the effect of the imaging gradient pulses [12]. Still, some colleagues (including prominent ones) yelled at me during my talks saying it was just not possible to measure diffusion in the brain, despite my efforts to explain that *incoherent* molecular motion and *coherent* macroscopic head motion could be sorted out. It was very discouraging, but, fortunately for diffusion MRI, I kept going and diffusion MRI progressively gained momentum. Nowadays there are several hundreds of oral and poster sessions dedicated to diffusion MRI methodology and applications, and the Diffusion Study Group is the largest by far within ISMRM.

## IVIM: From diffusion to perfusion

At the same time, I was working on the idea that diffusion MRI could also provide information on tissue perfusion with the goal to obtain functional images of brain activation in a similar way to PET, but without injection of tracers (see below). My initial intuition for liver angiomas was that blood movement in a complex tissue microvasculature could mimic a diffusion process on the macroscopic imaging scale and add-up a contribution to the true water diffusion process to the diffusion images. In a true (molecular) diffusion process, molecules move and collide with each other under the effect of their own thermal energy. Each collision alters the direction of motion of each molecule, and the overall process is well described by the random walk process described by Einstein [4]. I conjectured that, in addition to molecular diffusion, although on a completely different scale, the pattern of packets of flowing blood water molecules changing direction several times between each successive capillary segment could be described as a *pseudo-diffusion* process, given that these segments are arranged in space in a quasi-random way. The overall motion of those packets resembles a random walk, and Einstein’s mathematical model of diffusion should work here too. Still, there is huge difference in spatial scale between the elementary distances involved with genuine molecular diffusion processes (nanometers) and the capillary segment length at the origin of this blood microcirculation driven pseudo-diffusion process (tens of micrometers). However, Einstein’s diffusion/pseudo-diffusion coefficients combine the effects of both elementary particle velocity and distance: molecular diffusion is a very fast process occurring on molecular distances, while pseudo-diffusion in the blood stream occurs on much larger distances, but at a much slower speed. Overall, I found that tissue water diffusion and blood water pseudo-diffusion coefficients would differ by only about one order of magnitude (the molecular diffusion coefficient D for water in tissue is around 1 10^−3^mm^2^/s, while the pseudo-diffusion coefficient D* associated with blood capillary flow is around 10 10^−3^mm^2^/s in the brain). This proximity between D and D* values fully confirmed my intuition that MR diffusion images reflect the effects of both tissue diffusion and blood microcirculation, particularly with the very low b values I used at the time. Hence, in vivo the outcome of diffusion MRI was not the true diffusion coefficient of water in tissues, but contained some contribution from blood microcirculation. I decided to call the outcome parameter of diffusion MRI an *Apparent Diffusion Coefficient* (ADC)^*^, and this acronym entered history. After some brainstorming I came up with the IVIM (IntraVoxel Incoherent Motion) concept, to cover all random molecular displacements to which “diffusion” MRI could be sensitive, notably diffusion and blood microcirculation, but not only (see below for a recent addition to the IVIM family with virtual MR elastography). Hereafter, I published in 1986 my first article on diffusion MRI in the *Radiology* journal [13] with the title “Imaging of intravoxel incoherent motions: application to diffusion and perfusion in neurologic disorders”, showing the very first clinical brain diffusion MR images obtained in patients (Fig. 1). This seminal paper has been cited to date about 5000 times and is the most cited paper published in *Radiology* since its origin in 1923 for the neuroradiology domain [14].^1^

**Fig. 2:**
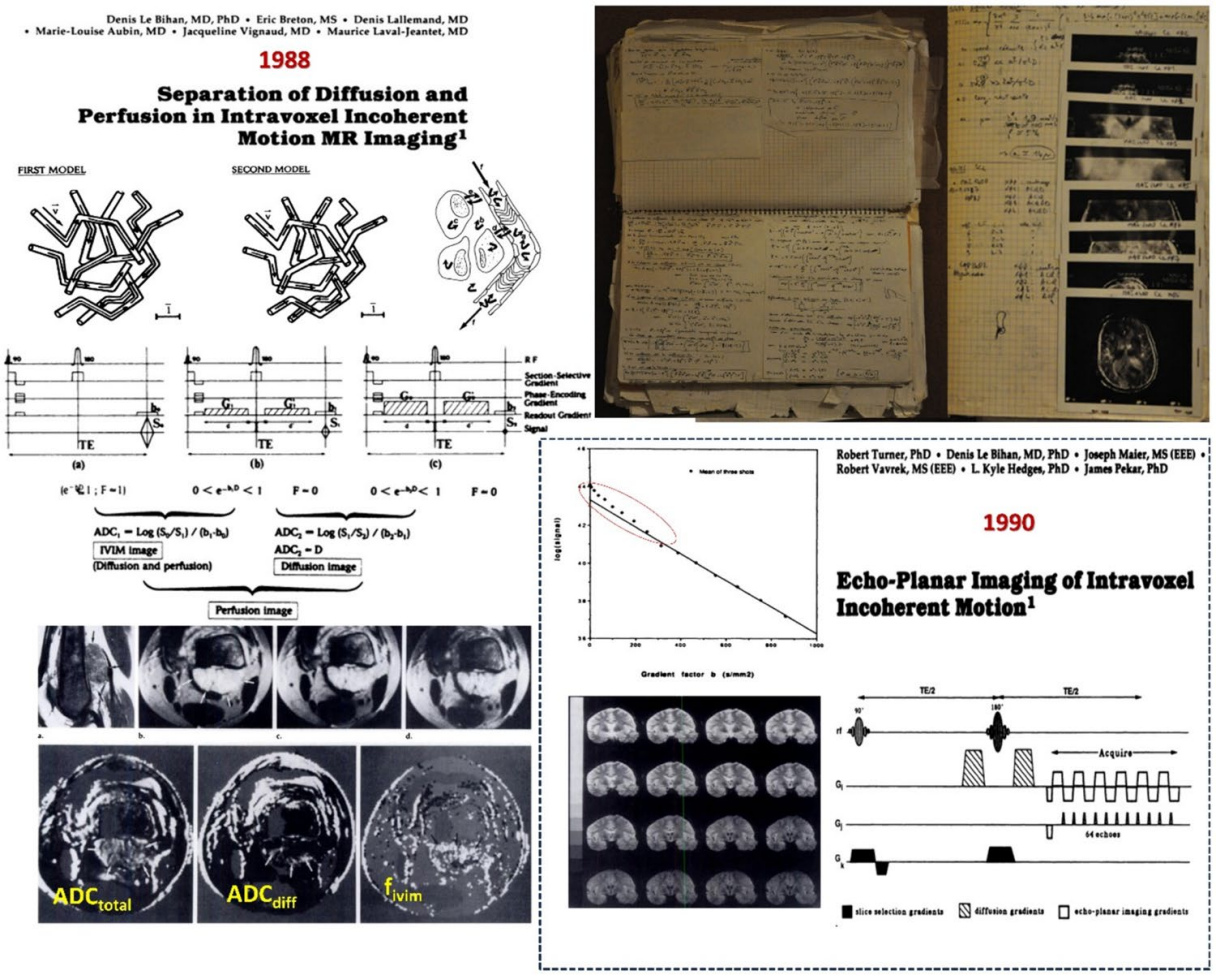
IVIM MRI. Left: The basic principles underlying the pseudo-diffusion model linked to blood microcirculation were first shown in an article published in Radiology in 1988 [15]. Two models were introduced, depending on the existence or not of direction changes in the blood flow during the encoding time. It was also shown how perfusion-driven IVIM effects can be separated from tissue diffusion effects to produce “perfusion faction” fIVIM maps from images acquired with 3 different b values. An example was shown in a malignant tumor of the femur. Top right: Extract from the notebook of the author showing the development of the IVIM theory and the first clinical examples obtained in patients (1986). Bottom right: First report of the use of Echo-Planar Imaging (EPI) to obtain IVIM and diffusion MR images [22]. Acquisition times were drastically shortened (16 slices in less than 1 min), producing images virtually free from motion artifacts. The strong gradient hardware required for EPI also benefited diffusion MRI by allowing much larger b values to be reached (here about 1000 s/mm^2^). Perfusion-driven IVIM effects were clearly visible as a curvature in the signal decay at very low b values

But this proximity between D and D* also meant that separation of pseudo-diffusion from diffusion effects could be achieved, so that diffusion MRI could also be used as a method to get *perfusion* images. The theoretical framework for perfusion imaging using the IVIM concept and the demonstration of the validity of this concept in phantoms and in vivo were exposed in a Scientific Exhibit at the next RSNA meeting in 1986 (which was awarded a Magna Cum Laude), also making the topic of my second seminal *Radiology* paper [15] (Fig. 2). This article which has been cited to date near 4000 times, making it the second most cited paper published in *Radiology* since 1923 for neuroradiology [14] was accompanied by a terrific editorial by Thomas Dixon [16]. Tom, now deceased, kindly gave me many years later a copy of the review he had sent to *Radiology* for my article. I was shaken by his laudatory comments, as I had no idea my work had received such recognition from the beginning. Indeed, to my dismay, perfusion-driven IVIM MRI which was a completely new concept coming out of the blue (Einstein’s equations to model perfusion) was not well received at the start, with colleagues questioning the validity of the concepts. It is true that separating perfusion-driven IVIM effects from diffusion MRI images was relatively challenging, technically, requiring a good signal-to-noise ratio and stable gradient material, which was difficult to achieve on the low-field MRI systems at the time. Furthermore, conceptually, “perfusion” as observed in IVIM MRI corresponds in fact more to the radiologist perspective (blood flow in the vascular compartment) than the physiologic perspective (blood flow to supply tissue, as measured, for instance, with PET), a point which I had to clarify, showing that the IVIM framework could, actually, bridge those two anatomic and physiologic conceptual definitions [17].

Because of my IVIM article and this editorial, diffusion MRI has been associated for many years with perfusion imaging, hence the many “diffusion/perfusion” sessions at meetings and workshops, or even book chapters, or journal key words, although they refer to completely different phenomena, both physically and biologically. Indeed, the precise properties measured by IVIM MRI have been the subject of countless doctoral theses, dissertations and a recent textbook [18], even dedicated ISMRM workshops, the most recent one (February 2024) in Erlangen, Germany. IVIM MRI is now experiencing a remarkable resurgence, particularly in oncology where repeated MRI scans may be necessary, as quantitative perfusion images can be obtained without the use of contrast agents, a concern in some patient populations at risk (eg, developping Nephrogenic System Fibrosis, NSF) when using gadolinium-based contrast agents (GBCA), the occurence of gadolinium deposits after repeated MRI exams, notably in the brain [19]), as well as for the impact of gadolinium on the environment [20]. Hence, IVIM MRI also appears as a method of choice for studies in pediatric populations, in the fetus or the placenta.

## From bench to bedside

Diffusion MRI received a great deal of attention following an unexpected discovery by Michael Moseley and his team at the University of California, San Francisco (UCSF). The team had been using diffusion MRI (actually, IVIM) to study perfusion in a model of acute cerebral ischemia in cats, based on middle cerebral artery occlusion. However, their main finding was the disconcerting reduction in the tissue diffusion coefficient of water that occurs in the early stages of acute cerebral ischemia, while standard MRI (T1, T2) was completely normal [21]. This breakthrough gave a major boost to diffusion MRI, which was still a puzzling laboratory tool, by attracting clinicians and persuading manufacturers to ensure that diffusion imaging was implemented in systems intended for clinical applications. Indeed, until the advent of diffusion MRI there was no definite diagnosis for patients undergoing an acute brain stroke caused by a clot blocking an artery in the brain. Differential diagnosis between a clot and a hemorrhage was more a guess, and there was no treatment available at all. Nearly half of the patients would die and the other would stay with permanent handicaps, such as being hemiplegic or aphasic for life. The social burden, the economic cost, everything was—and still is, in fact—considerable.

Clearly, diffusion MRI changed forever the destiny of acute brain stroke patients. Yet, for diffusion MRI to enter the clinical field the main issue was first to drastically reduce the acquisition time (about 10 min for each b value at the time) and the occurrence of motion artifacts. Everything changed with my collaboration with Robert Turner, who joined the National Institutes of Health (NIH) shortly after me. His unique expertise in gradient hardware and echo-planar imaging (EPI). Robert came from the famous Notthingham laboratory of Peter Mansfield, the father of EPI, enabled us to obtain the first IVIM-EPI images [22]. EPI is a “single-shot” imaging technique, which virtually “freezes” motion, as IVIM and artifact-free diffusion images could now be obtained in a matter of seconds (Fig. 2). The implementation required the making of a prototype dedicated gradient coil and the installation of power supplies by GEMS that were very powerful for their time. That move made IVIM MRI to clinically take off on commercial systems, first in the hand of GEMS through some of their skilled engineers who worked with us at the NIH. Benefiting from this advance, some colleagues from Harvard could confirm for the first time in patients that using EPI-diffusion MRI they could reproduce the results obtained at UCSF in the cat model [23, 24]: Within the first hours after the acute stroke a bright area corresponding to the area where neurons were suffering and dying was visible on the diffusion-weighted MR images while standard (T1, T2) MR images remained completely normal. The possibility to establish an accurate diagnosis was a breakthrough, making it possible for pharmaceutical companies to launch clinical trials on thrombolytic agents to dissolve the clots in vessels. Overnight, it became possible to treat and save patients, providing that thrombolysis treatment was administered soon enough (within the first hours after the onset of the stroke): some hemiplegic or aphasic patients could recover instantaneously, escaping the terrible destiny that was lying ahead of them by remaining handicapped for the rest of their life. Diffusion MRI was the tool which started it all and permitted this revolution in the way to manage those patients. Their breakthrough pushed other MRI manufacturers to implement EPI and diffusion MRI on their systems. Subsequent advances in MRI technology followed, combining EPI with parallel imaging using multiple channels, reducing echo times, making acquisitions less vulnerable to motion, using respiratory-triggering, and benefiting from state-of-the art gradient hardware going above 40 m/T/m.

## The birth of the brain connectome

**Fig. 3:**
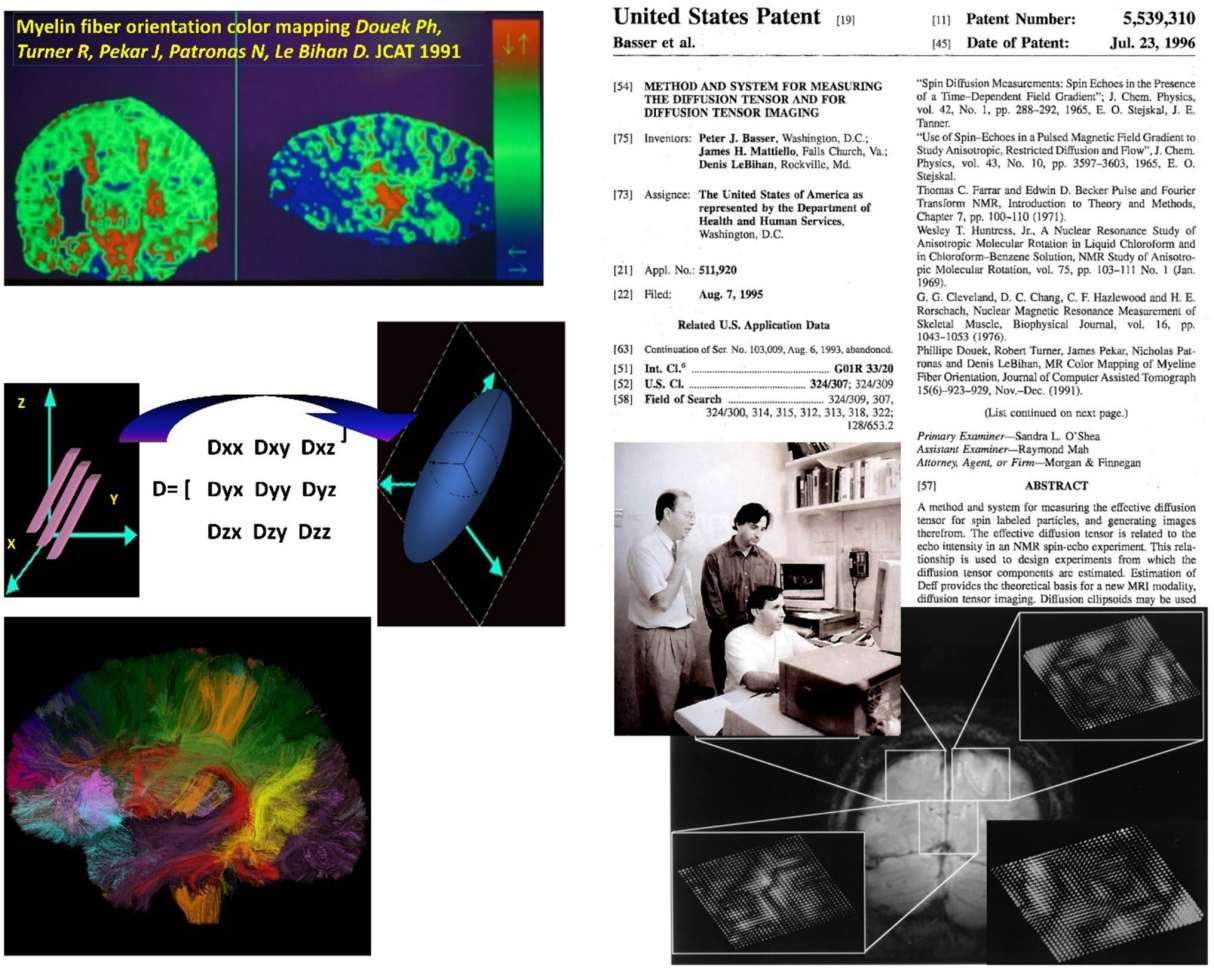
Diffusion Tensor Imaging (DTI). Top left: First color-coded images of white watter tracks orientation obtained with diffusion MRI (1991) [29]. The orientation is assumed to be aligned with the direction of highest diffusivity (here 2 directions were used). Right: Invention of DTI (Basser, Mattiello, Le Bihan shown on the photo). The DTI framework allows an accurate estimation of the fiber direction and of the diffusivity along all directions, which can be displayed as ellipsoids. Bottom left: DTI led to the development of tractography using specific algorithms to linked adjacent voxels and retrieve the trajectories of underlying white matter bundles (curtesy of C. Poupon, NeuroSpin)

Diffusion is truly a three-dimensional process, but water molecular mobility in tissues may not necessarily be the same in all directions. This diffusion *anisotropy* may result from the presence of obstacles that limit molecular movement in some directions more than others. Slight anisotropic diffusion effects were observed in excised biologic tissues in the 1970s in tissues with strongly oriented components, such rat skeletal muscles [25, 26]. The discovery from diffusion MRI that water diffusion was anisotropic in the central nervous system white matter (spinal cord then the brain) came as another breakthrough from the UCSF team around 1990 [27, 28], a period which I often qualify as the gold age of MRI. With diffusion MRI only the molecular displacement component along the magnetic field gradient direction used for diffusion sensitization is encoded at a time. By changing this direction, it was found that the ADC measured in the direction of the fibers was about 3–6 times higher than in the perpendicular direction. Diffusion anisotropy in white matter grossly originates from its specific organization in bundles of more or less myelinated axonal fibers running in parallel (see below). Immediately after the report of this discovery I thought about reverting this observation, a kind of reverse engineering, to determine and map the orientation of white-matter fibers in the brain: Assuming the direction of the fibers is parallel to the direction with the highest ADC, we can infer this orientation simply by estimating the ADC along multiple directions, setting up the founding principle which later underlined *tractography*. Our first attempt was very crude, using only two main directions and a simple color display as a proof of concept [29], but with this pioneering work, for the first time, we made white matter colorful, and obtained images of the orientation of the fiber bundles at each location: the germ of tractography and of the future stunning fiber tract 3D displays which now make the cover of journals or textbooks was seeded (Fig. 3).

During the “NIH Research Festival” of October 1990 I met Peter Basser who had a poster on ionic fluxes in tissues while I had a talk on our recent diffusion MRI results. Peter appropriately commented that the correct way to deal with anisotropic diffusion was to estimate the full diffusion *tensor*, not just the ADC, as the approach of the time provided. Basically, ADCs are not sufficient in the presence of diffusion anisotropy, except in particular cases where the main diffusion directions coincide with those of the diffusion MRI measurements. To solve this issue Peter and I came with a new paradigm, the Diffusion Tensor Imaging (DTI) framework (Fig. 3). By applying simultaneous diffusion-sensitizing gradient pulses along the X, Y and Z axes the diffusion MRI signal would become a linear combination of the diffusion tensor components. From the diffusion MRI signals acquired along a set of non-colinear directions, encoding multiple combinations of diffusion tensor components weighted by the corresponding b values, it would be possible to retrieve the individual diffusion tensor components at each location. As the diffusion tensor is symmetric in space, only 6 directions would be necessary. Those components are obtained in the X, Y, Z reference frame of the MRI scanner, but it is easy to find for each voxel location the reference frame (so-called “eigen-vectors”) coinciding, instead, to the orientation of the fibers trough an ad-hoc mathematical transformation (diagonalization), together with the “eigen-values” (*λ*) of the diffusion tensor which represent diffusivity along each of those local frame axes. The eigen-vector associated to the highest eigen-value corresponds to the predominent orientation of the fibers in each voxel. With DTI the “b factor” (scalar) had to be developed to a “b matrix” [30], an array of numbers to account for multiple cross-terms between diffusion coding and imaging gradient pulses not only along each axis, but also between perpendicular axes, a tedious task, especially with EPI, which our postdoc James Mattiello proudly handled [31]. Our first DTI attempts were made on vegetables and meat (pork muscle), then monkey brains before the first attempts on human brains. Our first account of DTI, setting up the principles, was published as another landmark paper in the Journal of Magnetic Resonance (4500 citations to date) [32], followed by another full fledge seminal article in the Biophysical Journal [33] (7500 citations), where we showed how the diffusion distance traveled by water in three-dimensional space during a given diffusion time could be represented by 3D ellipsoids (Fig. 3).

DTI can provide elaborate information on tissue microstructure and structure [34–37]. Basically, three types of information are obtained at the same time: The mean diffusivity, MD (similar to the isotropic ADC), which characterizes the global presence of obstacles to diffusion; the degree of anisotropy, which describes the asymmetry of the diffusion displacements in space (eccentricity of the ellipsoid) reflecting the presence of oriented structures. The standard marker is the fractional anisotropy (FA). Other quantities, such as the eigenvalue difference, λ_1_–λ_3_, should be avoided, as they are exquisitely sensitive to noise, depending on MD values, and may lead to the incorrect impression of the presence of anisotropy when there is none [38]. The third DTI parameter is, of course, the principal direction of diffusivity (principal elliptical axis), which is linked to the spatial orientation of the structure.

The next step toward “tractography” was to “connect” adjacent voxels on the basis of the orientation of each fiber tracks it contains in order to reconstruct fiber trajectories. Several algorithms were developed some years later [39–42]. A limitation of DTI is that it gives the global orientation of the white-matter bundles in each voxel based on the assumption that a single orientation predominates in each voxel. Significant steps were then made to resolve such a limitation (e.g., presence of fanning or crossings fibers) and improve the accuracy of tractography. Those approaches, so-called HARDI (High Angular Resolution Diffusion Imaging) methods [43], e.g., Q-ball [44] and DSI (Diffusion Spectrum Imaging) [45], have benefited from high angular resolution acquisition schemes (beyond the minimum of 6 required for basic DTI), and also from taking into account the contribution of intraaxial water through the use of very high b values. Other ways, such as stochastic approaches [46–48], have also been used to build for the first time atlases of the brain connections, the Connectome.

While DTI works remarkably well, one must recognize that the origin of the white-matter anisotropy is not entirely clear. Obviously, the directional restriction of water diffusion in axons (anisotropic restricted diffusion) contributes significantly to the overall anisotropy. However, although myelin certainly plays an important role in regulating the degree of anisotropy, as observed in demyelinating diseases, anisotropy is also observed in unmyelinated fibers (indeed, FA increases during brain maturation, as fibers become myelinated and increase action potential propagation speed [49]). The presence of anisotropy even in the absence of myelination, particularly in very dense and compact fiber bundles, may be explained by the parallel arrangement of fiber membranes in the bundles. Extracellular water also contributes to anisotropic effects, especially when diffusion weighting is low: perpendicular to the fibers, water molecules must diffuse along sinuous pathways around the fibers. Neurofilaments inside axons may also play a role. Overall, in addition to the crucial role of myelin, experimental data point to the importance of the spatial organization of membranes and intra-axonal content, even more so in gray matter. One way to address this issue if to investigate diffusion of molecules other than water, such as metabolites and neurotransmitters, which are clearly compartmentalized, through diffusion MR spectroscopy, as we showed for the first time in the human brain in 1993 [50].

## The social life of water

Following technical developments in MRI hardware (notably gradient systems) in the early 2000s diffusion MRI started to be used outside the brain. Diffusion MRI was shown to provide a new kind of contrast in oncology, allowing to diagnose and classify tumors and even to monitor treatment efficacy [1]. What is peculiar about diffusion MRI is that the physical process at play, diffusion, is a concept which has “a life on its own”, MRI being used merely as a method to image and measure it in vivo. This is not the case for the T1 and T2 relaxation parameters, for instance, which are meaningless outside the MRI context. MRI is, thus, only a tool to reveal the detailed mechanisms of diffusion processes in biologic tissues. Hence, it is legitimate and important to make further progress to understand what diffusion MRI images tell us. For example, what is the mechanism which leads to a sharp decrease in ADC acute brain ischemia? If it is related to cell volume expansion (cytotoxic edema), how does it slowdown diffusion exactly? Or what explains ADC decreases observed with the cell proliferation in malignant tissues? Many interesting models have been developed, unfortunately sometimes with experimental evidence to contradict them. For example, diffusion was thought to be faster in extracellular space than in intracellular space, which might explain why diffusion decreases with cell expansion or proliferation. However, it has been noted that the volume of intracellular space in the brain is at least 80% water, while the slow-diffusing component (if it represents the cellular compartment) accounts for at most a third of total water. What is more, some studies have reported that fast diffusion is also observed inside cells.

Tissues are complex organizational structures, and the diffusion process there considerably differs from the free Brownian motion modeled by Einstein. Diffusion-driven displacements do not follow anymore a Gaussian distribution, which becomes clearly visible when high b values are used: The signal decay is no longer a straight line, the slope of which is the diffusion coefficient, but get curved, the ADC becoming smaller. This behavior was invisible in the early days of diffusion MRI, as achievable b values were lower than 200 s/mm^2^. But in the 1990s, with the advent of the gradient hardware required for EPI, b values rose to 1000 s/mm^2^, today easily reaching 3000 s/mm^2^ (much higher in preclinical systems or with specially equipped clinical MRI scanners). While the ADC is often referred to as the “monoexponential”, Gaussian diffusion model, it is, indeed, sensitive to all diffusion behaviors, Gaussian and non-Gaussian (including pseudo-diffusion from blood microcirculation at very low b values, as we have seen). Due to the non-Gaussian diffusion curvature the ADC values depend on the choice of b values, which is both a pitfall (requiring standardization to compare results from different sites [38]) and a benefit (including extremely valuable information on tissue structure responsible for the non-Gaussian diffusion behavior). However, the quantitative characterization of such non-Gaussian diffusion effects requires the use of specific models. Some models have been introduced to deal empirically with the signal curvature, such as including polynomial or kurtosis models (also known as diffusion kurtosis imaging, DKI [51, 52], or the exponential stretch models (see [53] for a review). Although these models have led to the emergence of new parameters beyond ADC, such as the Kurtosis, and shown great potential for characterizing pathologic or physiologic conditions, they provide only “empirical” information on the degree of non-Gaussianity of diffusion, but no specific information on tissue characteristics. Recently, we introduced a model-free approach called signature index [1, 54] which, without the need to estimate model parameters, allows a direct classification of microscopic tissue features from signal behavior. Tissues features are identified by a direct comparison (distance calculation) of multi-b-value signal patterns with a library of known (or simulated) tissue signal signatures.

On the other hand, “explanatory” models are designed to provide estimates of physical parameters of tissue features, such as axonal diameters in brain white matter (CHARMED (Composite Hindered and Restricted Model of Diffusion) [55] and AxCaliber [56] models) or neurite orientation distributions (NODDI (Neurite Orientation and Dispersion and Density Imaging) model [57]). The diffusion time, which is unfortunately most of the time hidden to users of MRI systems, can be modulated to provide insightful information of the scaling of diffusion hindrance or restriction effects vary with time, leading, for instance, to estimates of cell size (VERDICT (Vascular, Extracellular, and Restricted Diffusion for Cytometry in Tumor) model [58], IMPULSED (Imaging Microstructural Parameters using Limited Spectrally Edited Diffusion) model [59–62]). Unfortunately, extracting such precise features from diffusion signals might be challenging due to the involvement of unknown factors to which diffusion MRI might be sensitive. Even if the basic diffusion mechanisms are known, a priori knowledge and strong assumptions about tissue structure (e.g., cell shape) must often be made to use those models appropriately and make accurate inferences about tissue characteristics. For instance, with 2-compartments models differences in compartmental relaxation effects are generally neglected. Furthermore, the cell membrane plays a central role as a barrier to water diffusion. However, most models share a common shortcoming: the influence of membrane permeability (in terms of water exchange and cell residence time) is most often largely ignored, for the simple reason that it is generally unknown. Yet, these effects can have a significant impact on the observed behavior of the diffusion signal, not to mention the estimated model parameters, especially when long diffusion times are used [63]. Oppositely, diffusion MRI might be used, instead, to estimate cell membrane permeability [64–66].

In addition to the “mechanical” or geometrical factors that can modulate the diffusion random walk of water, we must not forget that what actually makes the diffusion MRI signal is the diffusion movement of water hydrogen nuclei. Water is a small, highly polarizable molecule that doesn’t exist on its own, but belongs to a dense, “social” network of water molecules that interact via hydrogen bonds with other molecular structures such as polymers and proteins. Diffusing hydrogen nuclei constantly break and reconfigure these bonds when they propagate through the network. Indeed, water has a leading role in biology, from protein and membrane dynamics to cell physiology. In addition to linking proteins via hydrogen bonds, water can form molecular networks, and its properties (including diffusion) can change in the vicinity of charged cell membranes and intracellular membranes. I proposed a global diffusion model based on the physical structure of water in tissues and tried to complement the interpretation based solely on geometric compartments [67]. The amount of highly “structured” water in cells, especially near cell membranes, is itself a subject of great controversy between physicists and biologists, as shown in my book entitled “Water: the forgotten biomolecule” [68]. Given the large surface-to-volume ratio of most cells, these membrane-interacting water networks probably make up a significant proportion of tissue water. Consequently, variations in the shape, size and density of cells (or intracellular components) can lead to significant variations in membrane surface, which can affect the diffusion MRI signal. This new field of research, which explores as yet unchartered aspects of the role of water in biologic processes, should benefit greatly from diffusion MRI and shed light on its mechanisms.

**Fig. 4:**
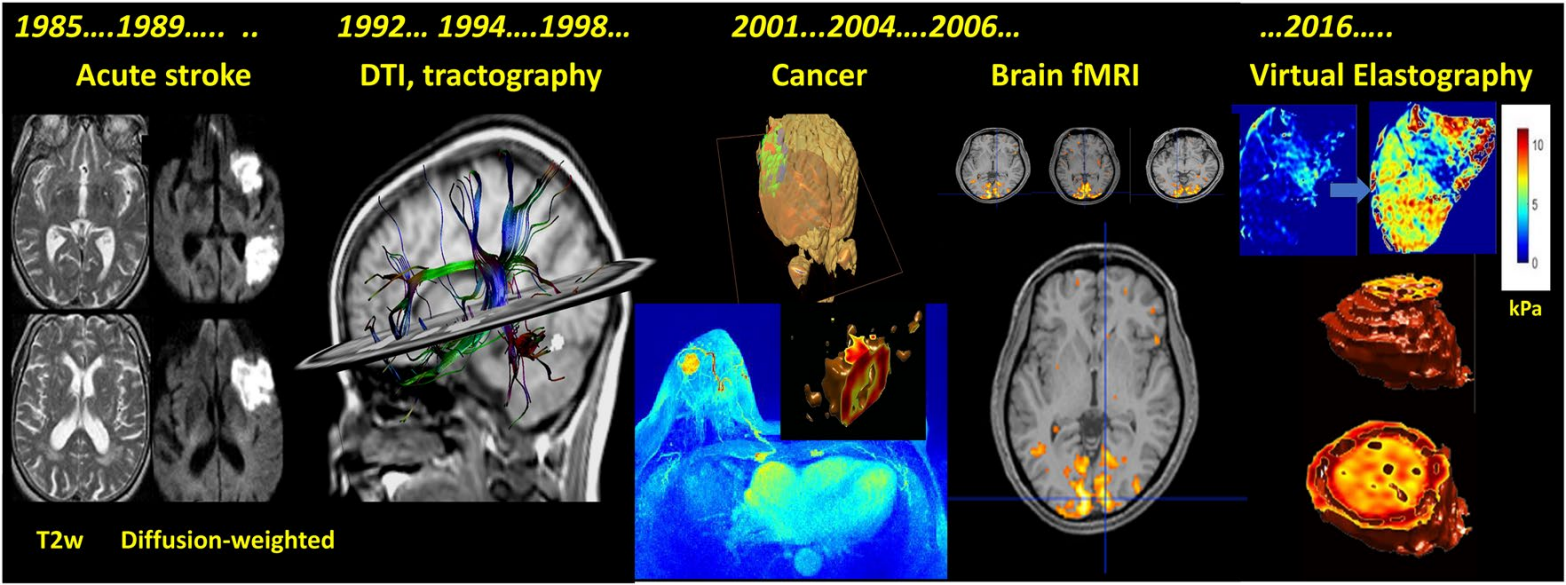
Historical development of clinical diffusion MRI. Following its introduction in 1985 diffusion MRI entered the clinical field when it was shown that diffusion MRI could, for the first time, show brain regions underlying acute brain ischemia which led to the development of thrombolytic treatments and completely changed overnight the fate of patients. In the 1990s DTI appeared, giving birth to tractography. Tractography is now used in neurosurgical planning and is becoming a key modality to investigate psychiatric disorders. In the 2000s progress in MRI hardware allowed diffusion and IVIM MRI to be used in the body as well, with important applications in oncology (diagnosis and treatment monitoring). It was also shown that diffusion MRI could be used to produce fMRI maps, as an alternative to BOLD fMRI, providing a more direct approach to the investigation of neuronal activity. Recently, it was also shown that the outcome of diffusion MR images could be translated into elasticity maps (virtual MR elastography, vMRE), providing quantitative estimates of tissue stiffness without the use of mechanical vibrations, of great interest for instance to stage liver fibrosis or investigate the nature of tumors

In addition, I have very recently shown that the stiffness of tissues, such as the liver, can be quantitatively estimated using diffusion MRI (so-called virtual MR Elastography of vMRE) [69–71] (Fig. 4). The reason is that microscopic features of tissues (such as the density of fibers and cells) have a similar effect on water diffusion, as seen with diffusion MRI, and the elastic properties of the tissue, as seen with MR elastography. Although this assumption needs to be validated across organs several groups have published amazing results, especially in the brain [72, 73]. A great feature of vMRE is that there is no need for the expensive and complex standard MRE hardware to generate mechanical vibrations. Knowing elasticity of tissues on a voxel basis though vMRE maps allows effects of propagating virtual mechanical waves of any frequency through the tissues to be simulated, with the resulting intravoxel phase-shift dispersion at the origin of a new kind of IVIM effect and contrast [69].

## Investigating brain function with IVIM, diffusion and DTI

On the first images obtained at the beginning of IVIM MRI a notable feature was that incoherent CSF flow in ventricular cavities was strikingly visible (Fig. 1). This observation led to the idea that IVIM MRI could be used to investigate CSF flow [74]. Unexpectedly, this idea resurfaced many years later, in the context of the glymphatic system, a recently introduced framework hypothesized to be a waste clearance in the brain through perivascular spaces [75], a kind of lymphatic system in the central nervous system. This concept might explain how the clearance failure of some macromolecules could lead to neurodegenerative disorders, such as Alzheimer’s disease, or the deposit of gadolinium in the brain from GBCA [19]. Astrocytes, which are present in the perivascular spaces, might play a key role through their aquaporin-4 (AQP-4) channels. Visualizing the glymphatic system clinically remains a challenge, however diffusion MRI has been shown to be sensitive to AQP-4 modulated astrocyte activity [76] and could, thus, be used as a marker of the glymphatic system, while IVIM might provide information on the brain interstitial fluid transport, instead of more invasive methods based on the injection of GBCA (see [77] for a review).

On the other hand, in the mid-1980s, one of my main goals in IVIM imaging was to obtain cerebral perfusion maps to study brain function, relying on the known links between neuronal activation, metabolism and blood flow (so-called neurovascular connections [78]) then exploited by PET. However, IVIM-based fMRI was no match when competing methods appeared in the early 1990s. After a nascent approach based on contrast agents [79], the concept of blood oxygenation level dependence (BOLD) quickly emerged as the leading approach [80]. BOLD fMRI was clearly easier to implement and more sensitive, so there was no room for the more difficult-to-implement IVIM method. However, subsequent studies have demonstrated the validity of the IVIM concept, as increased IVIM perfusion parameters in areas of brain activation can help us understand the contribution of individual vessels to the fMRI signal [81, 82]. IVIM MRI has also been used in conjunction with BOLD fMRI, as a kind of negative way, to improve the spatial resolution of BOLD fMRI by removing the flow contribution of the large arteries and veins that feed and drain large neuronal areas [82].

However, another promising avenue that has recently emerged is the possibility of using diffusion MRI to more directly detect brain activation instead of BOLD [83, 84] (Fig. 4). We are not referring here to IVIM or perfusion, but to the genuine diffusion changes that occur in tissue during neuronal activation. BOLD fMRI has been a great success for functional neuroimaging, but it has well-known limitations. The extent and mechanisms of the link between neuronal activation, metabolism and blood flow (neurovascular coupling) are not fully understood and may fail in pathologic conditions or in the presence of drugs, rendering BOLD fMRI not so reliable for clinical applications. Furthermore, the functional spatial resolution of vascular functional neuroimaging is ultimately limited because the blood vessels involved in increasing blood flow and volume supply and drain a rather large area containing groups of neurons with potentially different functions. Close neural circuits with different functions often share a common vasculature, especially on the venous side, which implies that there is a limit in the spatial resolution which can be obtained with BOLD fMRI [85], an issue with preclinical studies or with the coming ultrahigh field clinical systems reaching a resolution of 100–200 μm [86, 87]. Similarly, the physiologic delay required for the mechanisms triggering the vascular response to operate intrinsically limits the temporal resolution of BOLD fMRI. It is fair to say that BOLD fMRI has not well permeated the clinical field, even neurosurgery, and there is no clear clinical application in sight, to the contrary of DTI. DTI is now a major imaging modality which is widely used in neurology, neurosurgery (neurosurgical planning), and even now psychiatry: While MRI exams are usually normal as far as gray matter is regarded, it seems that most psychiatric disorders are apparently associated with anomalies of some white-matter tracks, a major breakthrough to understand the clinical expression (phenotype) of mental illnesses [88–93].

In contrast to vascular approaches, diffusion *f*MRI has the potential to reveal changes in the intrinsic physical properties of water associated with cerebral activation, which may be more closely related to neuronal activation mechanisms (there is an extensive literature on neuronal swelling associated with cellular depolarization [94, 95]): The cortex has around 8 10^8^ synapses/mm^3^, 20,000 synapses/neuron and dendrites of 10 mm/neuron or 400 m/mm^3^ in length), enabling improved spatial and temporal resolution. Several preclinical studies based on pharmacologic challenges to inhibit neurovascular coupling and cell expansion have confirmed that diffusion fMRI responses are distinguishable from BOLD fMRI responses and differ in their mechanisms. Diffusion fMRI responses are independent of neurovascular coupling and follow the activity status of neurons more closely and precisely than BOLD fMRI, especially under anesthesia or vasoactive drugs [96–99]. Indeed, since water homeostasis and movement undoubtedly play a central role in brain physiology (neurons, unlike astrocytes, do not express aquaporin channels and cannot regulate their volume), microstructural effects reflected in water diffusion behavior during activation may be an active component of the neuron activation process. What contribution do these rapid “mechanical” changes observed in tissue microstructure (such as contraction of dendritic spines [100]) make to brain tissue function? Since the laws of nature are often reversible, could neurons act as piezoelectric sensors that depolarize by “sensing” the movement of neighboring cells, making information transfer within the neural network a faster alternative to synaptic transmission? Here again, diffusion MRI could shed light to understand and exploit such mechanisms.

## Conclusion

Diffusion MRI, as its additions, DTI and IVIM MRI, has become a pillar of modern medical imaging with broad applications in both clinical and research settings, providing insights into tissue integrity and structural abnormalities. It allows to detect early changes in tissues that may not be visible with other imaging modalities. Diffusion imaging first revolutionized the management of acute cerebral ischemia by allowing diagnosis at an acute stage when therapies can still work, saving the outcomes of many patients. Diffusion imaging is today extensively used not only in neurology but also in oncology throughout the body for detecting and classifying various kinds of cancers, as well as monitoring treatment response at an early stage. The second major impact of diffusion imaging concerns the wiring of the brain, allowing to obtain non-invasively images in 3 dimensions of the brain connections. DTI has opened up new avenues of clinical diagnosis and research to investigate brain diseases, revealing for the first time how defects in white-matter track integrity could be linked to mental illnesses.

## Footnotes

1 *Note: As I later discovered, Tanner had already referred to "apparent diffusion coefficients", Da, but not in the context of a generalized framework describing incoherent microscopic motion. His “apparent diffusion" referred to the "relative diffusion" observed in tissues in relation to free diffusion due to restricted diffusion effects.*

## References

[1] Iima M, Le Bihan D. Clinical intravoxel incoherent motion and diffusion MR imaging: past, present, and future. Radiology. 2016;278(1):13–32. 26690990 10.1148/radiol.2015150244

[2] Barentsz JO, Richenberg J, Clements R, Choyke P, Verma S, Villeirs G, et al. ESUR prostate MR guidelines. Eur Radiol. 2012;22:746–57. 22322308 10.1007/s00330-011-2377-y PMC3297750

[3] Baltzer P, Mann RM, Iima M, et al. Diffusion-weighted imaging of the breast-a consensus and mission statement from the EUSOBI International breast diffusion-weighted imaging working group. Eur Radiol. 2020;30:1436–50. 31786616 10.1007/s00330-019-06510-3 PMC7033067

[4] Einstein A. Uber die von der molecularkinetischen Theorie der Wärme geforderte Bewegung von in ruhenden Flüssigkeiten suspendierten Teilchen. Ann Phys (Leipzig). 1905;17:549–69.

[5] Le Bihan D, Delannoy J, Levin RL. Temperature mapping with MR imaging of molecular diffusion: application to hyperthermia. Radiology. 1989;171(3):853–7. 2717764 10.1148/radiology.171.3.2717764

[6] Delannoy J, Le Bihan D, Hoult DI, Levin RL. Hyperthermia system combined with a magnetic resonance imaging unit. Med Phys. 1990;17(5):855–60. 2233572 10.1118/1.596477

[7] Stejskal EO, Tanner JE. Spin diffusion measurements: spin echoes in the presence of a time-dependent field gradient. J Chem Phys. 1965;42:288–92.

[8] Wesbey GE, Moseley ME, Ehman RL. Translational molecular self-diffusion in magnetic resonance imaging. II. Measurement of the self-diffusion coefficient. Investive Radiol. 1984;19:491–8. 10.1097/00004424-198411000-00005 6511256

[9] Le Bihan D, Breton E. Imagerie de diffusion in vivo par résonance magnétique nucléaire. C R Acad Sci Paris. 1985;301:1109–12.

[10] Yamada I, Aung W, Himeno Y, Nakagawa T, Shibuya H. Diffusion coefficients in abdominal organs and hepatic lesions: evaluation with intravoxel incoherent motion echo-planar MR imaging. Radiology. 1999;210:617–23. 10207458 10.1148/radiology.210.3.r99fe17617

[11] Merboldt KD, Hanicke W, Frahm J. Self-diffusion NMR imaging using stimulated echoes. J Magn Reson. 1985;64:479–86.

[12] Taylor DG, Bushell MC. The spatial mapping of translational diffusion coefficients by the NMR imaging technique. Phys Med Biol. 1985;30:345–9. 4001161 10.1088/0031-9155/30/4/009

[13] Le Bihan D, Breton E, Lallemand D, Grenier P, Cabanis E, Laval-Jeantet M. MR imaging of intravoxel incoherent motions: application to diffusion and perfusion in neurologic disorders. Radiology. 1986;161:401–7. 3763909 10.1148/radiology.161.2.3763909

[14] Anzai Y, Erl-Wagner B. Neuroradiology 2040: a glimpse into the future. Radiology. 2023;308(3): e231267. 10.1148/radiol.231267. 37750766 10.1148/radiol.231267

[15] Le Bihan D, Breton E, Lallemand D, Aubin ML, Vignaud J, Laval-Jeantet M. Separation of diffusion and perfusion in intravoxel incoherent motion MR imaging. Radiology. 1988;168:497–505. 3393671 10.1148/radiology.168.2.3393671

[16] Dixon WT. Separation of diffusion and perfusion in intravoxel incoherent motion MR imaging: a modest proposal with tremendous potential. Radiology. 1988;168:566–7. 3393682 10.1148/radiology.168.2.3393682

[17] Le Bihan D, Turner R. The capillary network: a link between IVIM and classical perfusion. Magn Reson Med. 1992;27:171–8. 1435202 10.1002/mrm.1910270116

[18] Le Bihan D, Iima M, Federau C, Sigmund ES, editors. Intravoxel incoherent motion (IVIM) MRI: principles and applications. Singapore: Pan Stanford Publishing; 2018.

[19] Kanda T, Fukusao T, Matsuda M, Toyoda K, Oba H, Kotoku J, et al. Gadolinium-based contrast agents accumulates in the brain even in subjects without severa renal dysfunction: evaluation of autopsy brains specimens with inductively coupled plasma mass spectroscopy. Radiology. 2015;276(1):228–32. 10.1148/radiol.2015142690. 25942417 10.1148/radiol.2015142690

[20] Brünjes R, Hofman T. Anthoponegic gadolinium in freshwater and drinking water systems. Water Res. 2020;182: 115966. 10.1016/j.watres.2020.115966. 32599421 10.1016/j.watres.2020.115966 PMC7256513

[21] Moseley M, Cohen Y, Mintorovitch J, Chileuitt L, Shimizu H, Kucharczyk J, et al. Early detection of regional cerebral ischemia in cats: comparison of diffusion-and T2-weighted MRI and spectroscopy. Magn Reson Med. 1990;14(2):330–46. 2345513 10.1002/mrm.1910140218

[22] Turner R, Le Bihan D, Maier J, Vavrek R, Hedges LK, Pekar J. Echo-planar imaging of intravoxel incoherent motions. Radiology. 1990;177:407–14. 2217777 10.1148/radiology.177.2.2217777

[23] Warach S, Chien D, Li W, Ronthal M, Edelman RR. Fast magnetic resonance diffusion-weighted imaging of acute human stroke. Neurology. 1992;42:1717–23. 1513459 10.1212/wnl.42.9.1717

[24] Chien D, Kwong KK, Gress DR, Buonanno FS, Buxton RB, Rosen BR. MR diffusion imaging of cerebral infarction in humans. Am J Neuroradiol. 1992;13:1097–102. 1636519 PMC8333580

[25] Hansen JR. Pulsed NMR study of water mobility in muscle and brain tissue. Biochim Biophys Acta. 1971;230:482–6. 5581279 10.1016/0304-4165(71)90177-2

[26] Cleveland GG, Chang DC, Hazelwood CF, Rorschach HE. Nuclear magnetic resonance measurement of skeletal muscle. Anisotropy of the diffusion coefficient of the intracellular water. Biophys J. 1976;16:1043–53. 963204 10.1016/S0006-3495(76)85754-2 PMC1334944

[27] Moseley ME, Cohen Y, Kucharczyk J. Diffusion-weighted MR imaging of anisotropic water diffusionin cat central nervous system. Radiology. 1990;176:439–46. 2367658 10.1148/radiology.176.2.2367658

[28] Chenevert TL, Brunberg JA, Pipe JG. Anisotropic diffusion within human white matter: demonstration with NMR techniques in vivo. Radiology. 1990;177:401–5. 2217776 10.1148/radiology.177.2.2217776

[29] Douek P, Turner R, Pekar J, Patronas N, Le Bihan D. MR color mapping of myelin fiber orientation. J Comput Assist Tomogr. 1991;15(6):923–9. 1939769 10.1097/00004728-199111000-00003

[30] Mattiello J, Basser P, Le Bihan D. Analytical expressions for the b matrix in NMR diffusion imaging and spectroscopy. J Magn Reson Ser A. 1994;108:131–41.

[31] Mattiello J, Basser P, Le Bihan D. The b matrix in diffusion tensor echo-planar imaging. Magn Reson Med. 1997;37:292–300. 9001155 10.1002/mrm.1910370226

[32] Basser PJ, Mattiello J, Le Bihan D. Estimation of the effective self-diffusion tensor from the NMR spin echo. J Magn Reson. 1994;103:247–54. 10.1006/jmrb.1994.1037 8019776

[33] Basser PJ, Mattiello J, Le Bihan D. MR diffusion tensor spectroscopy and imaging. Biophys J. 1994;66:259–67. 8130344 10.1016/S0006-3495(94)80775-1 PMC1275686

[34] Pierpaoli C, Jezzard P, Basser PJ, Barnett A, DiChiro G. Diffusion tensor MR imaging of the human brain. Radiology. 1996;201:637–48. 8939209 10.1148/radiology.201.3.8939209

[35] Basser P. Inferring microstructural features and the physiological state of tissues from diffusion-weighted images. NMR Biomed. 1995;8(7):333–44. 8739270 10.1002/nbm.1940080707

[36] Le Bihan D. Molecular diffusion, tissue microdynamics and microstructure. NMR Biomed. 1995;8(7):375–86. 8739274 10.1002/nbm.1940080711

[37] Le Bihan D, Mangin JF, Poupon C, Clark CA, Pappata S, Molko N, Chabriat H. Diffusion tensor imaging: concepts and applications. J Magn Reson Imaging. 2001;13(4):534–46. 11276097 10.1002/jmri.1076

[38] Iima M, Partridge SC, Le Bihan D. Six DWI questions you always wanted to know but were afraid to ask: clinical relevance for breast diffusion MRI. Eur Radiol. 2020;30:2561–70. 10.1007/s00330-019-06648-0. 31965256 10.1007/s00330-019-06648-0

[39] Poupon C, Mangin JF, Frouin V, Regis J, Poupon F, Le Bihan D, Bloch I. Regularization of MR diffusion tensor maps for tracking brain white matter bundles. In: Medical Image Computing and Computer-Assisted Intervention—MICCAI, 1998; Oct. 98. LNCS-1496, MIT, Springer-Verlag, pp. 489–498

[40] Conturo TE, Lori NF, Cull TS, Akbudak E, Snyder AZ, Shimony JS, McKinstry RC, Burton H, Raichle ME. Tracking neuronal fiber pathways in the living human brain. PNAS. 1999;96:10422–7. 10468624 10.1073/pnas.96.18.10422 PMC17904

[41] Mori S, Crain BJ, Chacko VP, Van Zijl PCM. Three-dimensional tracking of axonal projections in the brain by magnetic resonance imaging. Ann Neurol. 1999;45:265–9. 9989633 10.1002/1531-8249(199902)45:2<265::aid-ana21>3.0.co;2-3

[42] Jones DK, Simmons A, Williams SC, Horsfield MA. Non-invasive assessment of axonal fiber connectivity in the human brain via diffusion tensor MRI. Magn Reson Med. 2000;42:37–41. 10.1002/(sici)1522-2594(199907)42:1<37::aid-mrm7>3.0.co;2-o 10398948

[43] Frank LR. Anisotropy in high angular resolution diffusion-weighted MRI. Magn Reson Med. 2002;45:935–9. 10.1002/mrm.1125 11378869

[44] Tournier JD, Calamante F, Gadian DG, Connelly A. Direct estimation of the fiber orientation density function from diffusion-weighed MRI data using spherical deconvolution. Neuroimage. 2004;23:1176–85. 15528117 10.1016/j.neuroimage.2004.07.037

[45] Wedeen VJ, Hagmann P, Tseng WYI, Reese TG, Weisskoff RM. Mapping complex tissue architecture with diffusion spectrum magnetic resonance imaging. Magn Reson Med. 2005;54:1377–86. 16247738 10.1002/mrm.20642

[46] Behrens TEJ, Woolrich MW, Jenkinson M, JohansenBerg H, Nunes RG, Clare S, Matthews PM, Brady JM, Smith SM. Characterization and propagation of uncertainty in diffusion-weighted MR imaging. Magn Reson Med. 2003;50:1077–88. 14587019 10.1002/mrm.10609

[47] Hagmann P, Thiran JP, Jonasson L, Vandergheynst P, Clarke S, Maeder P, Meuli R. DTI mapping of human brain connectivity: statistical fibre tracking and virtual dissection. Neuroimage. 2003;19:545–54. 12880786 10.1016/s1053-8119(03)00142-3

[48] Parker GJ, Haroon HA, Wheeler-Kingshott CA. A framework for a streamline-based probabilistic index of connectivity (PICo) using a structural interpretation of MRI diffusion measurements. J Magn Reson Imaging. 2003;18:242–54. 12884338 10.1002/jmri.10350

[49] Dubois J, Hertz-Pannier L, Dehaene-Lambertz G, Cointepas Y, Le Bihan D. Assessment of the early organization and maturation of infants’ cerebral white matter fiber bundles: a feasibility study using quantitative diffusion tensor imaging and tractography. Neuroimage. 2006;30(4):1121–32. 16413790 10.1016/j.neuroimage.2005.11.022

[50] Posse S, Cuenod CA, Le Bihan D. Human brain: proton diffusion MR spectroscopy. Radiology. 1993;188(3):719–25. 8351339 10.1148/radiology.188.3.8351339

[51] Chabert S, Meca C, Le Bihan D. Relevance of the information about the diffusion distribution in vivo given by kurtosis in q-space imaging. Proceedings of the 12th Annual Meeting of ISMRM, 2004, Kyoto, Japan, 1238

[52] Jensen JH, Helpern JA, Ramani A, Lu H, Kaczynski K. Diffusional kurtosis imaging: the quantification of non-gaussian water diffusion by means of magnetic resonance imaging. Magn Reson Med. 2005;53(6):1432–40. 15906300 10.1002/mrm.20508

[53] Yablonskiy DA, Sukstanskii AL. Theoretical models of the diffusion weighted MR signal. NMR Biomed. 2010;3:661–81. 10.1002/nbm.1520 PMC6429954 20886562

[54] Goto M, Le Bihan D, Yoshida M, Sakai K, Yamada K. Adding a model-free diffusion MRI marker to BI-RADS assessment improves specificity for diagnosing breast lesions. Radiology. 2019;292:84–93. 31112086 10.1148/radiol.2019181780

[55] Assaf Y, Basser PJ. Composite hindered and restricted model of diffusion (CHARMED) MR imaging of the human brain. Neuroimage. 2005;27(1):48–58. 15979342 10.1016/j.neuroimage.2005.03.042

[56] Assaf Y, Blumenfeld-Katzir T, Yovel Y, Basser PJ. AxCaliber: a method for measuring axon diameter distribution from diffusion MRI. Magn Reson Med. 2008;59(6):1347–54. 18506799 10.1002/mrm.21577 PMC4667732

[57] Zhang H, Schneider T, Wheeler-Kingshott CA, Alexander DC. NODDI: practical in vivo neurite orientation dispersion and density imaging of the human brain. Neuroimage. 2012;61(4):1000–16. 22484410 10.1016/j.neuroimage.2012.03.072

[58] Panagiotaki E, Walker-Samuel S, Siow B, Johnson SP, Rajkumar V, Pedley RB, et al. Noninvasive quantification of solid tumor microstructure using VERDICT MRI. Cancer Res. 2014;74:1902–12. 24491802 10.1158/0008-5472.CAN-13-2511

[59] Jiang X, Li H, Xie J, MvKinley ET, Zhao P, Gore JC, Xu J. In vivo imaging of cancer size and cellularity using temporal diffusion spectroscopy. Magn Reson Med. 2017;78(1):156–64. 27495144 10.1002/mrm.26356 PMC5293685

[60] Jiang X, Xu J, Gore JC. Mapping hepatocyte size in vivo using temporal diffusion spectroscopy MRI. Magn Reson Med. 2020;84:2671–83. 32333469 10.1002/mrm.28299 PMC7402009

[61] Xu J, Jiang X, Li H, Arlinghaus LR, McKinley ET, Devan SP, et al. Magnetic resonance imaging of mean cell size in human breast tumors. Magn Reson Med. 2020;83:2002–14. 31765494 10.1002/mrm.28056 PMC7047520

[62] Wu D, Jiang K, Li H, Zhang Z, Ba R, Zhang Y, et al. Time-dependent diffusion MRI for quantitative microstructural mapping of prostate cancer. Radiology. 2022;303:578–87. 35258368 10.1148/radiol.211180

[63] Li H, Jiang X, Xie J, McIntyre JO, Gore JC. Time-dependant influence of cell membrane permeability on MR diffusion measurements. Magn Reson Med. 2016;75(5):1927–34. 26096552 10.1002/mrm.25724 PMC4676747

[64] Imae T, Shinohara H, Sekino M, Ueno S, Ohsaki H, Mima K, Ohtomo K. Estimation of cell membrane permeability and intracellular diffusion coefficient of human gray matter. Magn Reson Med Sci. 2009;8(1):1–7. 19336983 10.2463/mrms.8.1

[65] Obata T, Kershaw J, Tachibana Y, Miyauchi T, Abe Y, Shibata S. Comparison of diffusion-weighted MRI and anti-Stokes Raman scattering (CARS) measurements of the intercompartmentak exchange-time of water un expression-controlled aquaporin-4 cells. Sci Rep. 2018;8(1):17954. 10.1038/s41598-018-36264-9. 30560905 10.1038/s41598-018-36264-9 PMC6298983

[66] Gardier R, Haro JLV, Canales-Rodriguez EJ, Lesescu IO, Girard G, Rafael-Patino J, Thiran JP. Cellular exchange imaging (CEXI): evaluation of a diffusion model including water exchange in cells using numerical phantoms of permeable spheres. Magn Reson Med. 2023;90:1625–40. 37279007 10.1002/mrm.29720

[67] Le Bihan D. The, “wet mind”: water and functional neuroimaging. Phys Med Biol. 2007;52:R57–90. 17374909 10.1088/0031-9155/52/7/R02

[68] Le Bihan D, Fukuyama H, editors. Water: The forgotten biological molecule. Singapore: Pan Stanford Publishing; 2010.

[69] Le Bihan D, Ichikawa S, Motosugi U. Diffusion and intravoxel incoherent motion MR imaging-based virtual elastography: a hypothesis-generating study in the liver. Radiology. 2017;285(2):609–19. 28604279 10.1148/radiol.2017170025

[70] Kromrey ML, Le Bihan D, Ichikawa S, Motosugi U. Diffusion-weighted MRI-based virtual elastography for the assessment of liver fibrosis. Radiology. 2020;295(1):127–35. 32043948 10.1148/radiol.2020191498

[71] Ota T, Hori M, Le Bihan D, Fukui H, Onishi H, Nakamoto A, et al. Diffusion-based virtual mr elastograpgy of the liver: can it be extended neyond liver fibrosis? J Clin Med. 2021;10(19):4553. 10.3390/jcm101994553. 34640568 10.3390/jcm10194553 PMC8509260

[72] Lagerstrand K, Gaedes N, Eriksson S, Farahmand D, De Coursey E, Johansson G, et al. Virtual magnetic resonance elastography has the feasibility to evaluate preoperative pituitary adenoma consistency. Pituitary. 2021;24:530–41. 33555485 10.1007/s11102-021-01129-4 PMC8270838

[73] Aunan-Diop JS, Andersen MCS, Friismose AI, Halle B, Pedersen CB, Mussman B, et al. Virtual magnetic resonance elastography predicts the intreaoperative consistency of meningiomas. J Neuroradiol. 2023;50(4):396–401. 36343849 10.1016/j.neurad.2022.10.006

[74] Le Bihan D, Breton E, Aubin ML, Lallemand D, Vignaud J. Study of cerebrospinal fluid dynamics by MRI of intravoxel incoherent motions (IVIM). J Neuroradiol. 1987;14(4):388–95. 3450800

[75] Iliff JJ, Wang M, Liao Y, Plogg BA, Peng W, Gundersen GA, Benveniste H, et al. A paravascular pathway facilitates CSF flow through the brain parenchyma and the clearance of interstitial solutes, including amyloid b. Sci Transl Med. 2012;4:147ra111. 22896675 10.1126/scitranslmed.3003748 PMC3551275

[76] Debaker C, Djemai B, Ciobanu L, Tsurugizawa T, Le Bihan D. Diffusion MRI reveals in vivo and non-invasively changes in astrocyte function induced by an aquaporin-4 inhibitor. PLoS ONE. 2020;15(5): e0229702. 10.1371/journal.pone.0229702. 32413082 10.1371/journal.pone.0229702 PMC7228049

[77] Naganawa S, Taoka T. The glymphatic system: a review of the challenges in visualizing its structure and function with MR imaging. Mag Reson Med Sci. 2022;21(1):182–94. 10.2463/mrms.rev.2020-0122 PMC9199971 33250472

[78] Roy CW, Sherrington CS. On the regulation of the blood supply of the brain. J Physiol. 1890;11:85–108. 16991945 10.1113/jphysiol.1890.sp000321 PMC1514242

[79] Belliveau JW, Kennedy DN, McKinstry RC, Burchbinder BR, Weisskoff RM, Cohen MS, Vevea JM, Brady TJ, Rosen BR, Buchbinder BR. Functional mapping of the human visual cortex by magnetic resonance imaging. Science. 1991;254:716–9. 1948051 10.1126/science.1948051

[80] Ogawa S, Tank DW, Menon RS, Ellerman JM, Kim SG, Merkle H, Ugurbil K. Intrinsic signal changes accompanying sensory stimulation—functional brain mapping with magnetic resonance imaging. PNAS. 1992;89:5951–5. 1631079 10.1073/pnas.89.13.5951 PMC402116

[81] Song AW, Wong EC, Tan SG, Hyde JS. Diffusion weighted fMRI at 1.5 T. Magn Reson Med. 1996;35:155–8. 8622577 10.1002/mrm.1910350204

[82] Song AW, Bruce I, Petty C, Chen NK. IVIM fMRI: brain activation with a high spatial specificity and resolution. In: Le Bihan D, Iima M, Federau C, Sigmund ES, editors. Intravoxel Incoherent Motion (IVIM) MRI: Principles and Applications. Singapore: Pan Stanford Publishing; 2018.

[83] Darquie A, Poline JB, Poupon C, Saint-Jalmes H, Le Bihan D. Transient decreasein water diffusion observed in human occipital cortexduring visual stimulation. PNAS. 2001;98:9391–5. 11459931 10.1073/pnas.151125698 PMC55431

[84] Le Bihan D, Urayama S, Aso T, Hanakawa T, Fukuyama H. Direct and fast detection of neuronal activation in the human brain with diffusion MRI. PNAS. 2006;103:8263–8. 16702549 10.1073/pnas.0600644103 PMC1472461

[85] Sirotin YB, Das A. Anticipatory haemodynamic signals in sensory cortec not predicted by local neuronal activity. Nature. 2009;457:475–9. 19158795 10.1038/nature07664 PMC2705195

[86] Le Bihan D. Schild T Human brain MRI at 500MHz, scientific perspectives and technological challenges. Supercond Sci Technol. 2017;30:033003.

[87] Boulant N, Mauconduit F, Gras V, Amadon A, Le Ster C, Luong M, et al. First in vivo images of the human brain revealed with the Iseult 11.7T MRI scanner. Preprint available at Research Square. 10.21203/rs.3.rs-3931535/v1.

[88] Wolff J, Gu H, Gerig G, Elison JT, Styner M, Gouttard S, et al. Differences in white matter fibert tract development present from 6 to 24 months in infants with autism. J Psychiatry. 2012;169(6):589–600. 10.1176/appi.ajp.2011.11091447 PMC3377782 22362397

[89] Kelly S, Jahanshad N, Zalesky A, Kochunov P, Agartz I, Alloza C, et al. Widespread white matter microstructural differences in schizophrenia across 4322 individuals: results from the ENIGMA schizophrenia DTI working group. Mol Psychiatry. 2018;23:1261–9. 29038599 10.1038/mp.2017.170 PMC5984078

[90] Le Bihan D. On time and space in the brain: a relativistic pseudo-diffusion framework. Brain Multiphys. 2020;1: 100016. 10.1016/j.brain.2020.100016.

[91] Berkovitch L, Charles L, Del Cul A, Hamndani N, Delavest M, Sarrazin S, et al. Disruption of conscious access in psychosis is associated with altered structural brain connectivity. J Neurosci. 2021;41:513–23. 33229501 10.1523/JNEUROSCI.0945-20.2020 PMC7821858

[92] Raven EP, Veraart J, Kievit RA, Genc S, Ward IL, Hall J, et al. In vivo evidence of microstructural hypoconnectivity of brain white matter in 22q11.2 deletion syndrome. Mol Psychiatry. 2023;28:4342–52. 10.1038/s41380-023-02178-w. 37495890 10.1038/s41380-023-02178-w PMC7615578

[93] Le Bihan D. From black hole entropy to consciousness: the dimensions of the brain connectome. Entropy. 2023;25:1645. 10.3390/e25121645. 38136525 10.3390/e25121645 PMC10743094

[94] Andrew RD, MacVicar BA. Imaging cell volume changes and neuronal excitation in the hippocampal slice. Neuroscience. 1994;62:371–83. 10.1016/0306-4522(94)90372-7. 7830884 10.1016/0306-4522(94)90372-7

[95] Cohen LB, Keynes RD, Hille B. Light scattering and birefringence changes during nerve activity. Nature. 1968;218:438–41. 10.1038/218438a0. 5649693 10.1038/218438a0

[96] Abe Y, Tsurugizawa T, Le Bihan D. Water diffusion closely reveals neural activity status in rat brain loci affected by anesthesia. PLoS Biol. 2017;15:1–24. 10.1371/journal.pbio.2001494. 10.1371/journal.pbio.2001494 PMC5390968 28406906

[97] Abe Y, Van Nguyen K, Tsurugizawa T, Ciobanu L, Le Bihan D. Modulation of water diffusion by activation-induced neural cell swelling in *Aplysia californica*. Sci Rep. 2017;7:1–8. 10.1038/s41598-017-05586-5. 28733682 10.1038/s41598-017-05586-5 PMC5522485

[98] Tsurugizawa T, Ciobanu L, Le Bihan D. Water diffusion in brain cortex closely tracks underlying neuronal activity. PNAS. 2013;110(28):11636–41. 10.1073/pnas.1303178110. 23801756 10.1073/pnas.1303178110 PMC3710802

[99] Tsurugizawa T, Abe Y, Le Bihan D. Water apparent diffusion coefficient correlates with gamma oscillation of local field potentials in the rat brain nucleus accumbens following alcohol injection. J Cereb Blood Flow Metab. 2017;37:3193–202. 10.1177/0271678X16685104. 28058981 10.1177/0271678X16685104 PMC5584694

[100] Crick F. Do dendritic spines twitch? Trends Neurosci. 1982;5:44–6.

